# Interest of Monitoring Diaphragmatic Electrical Activity in the Pediatric Intensive Care Unit

**DOI:** 10.1155/2013/384210

**Published:** 2013-02-21

**Authors:** Laurence Ducharme-Crevier, Geneviève Du Pont-Thibodeau, Guillaume Emeriaud

**Affiliations:** Pediatric Intensive Care Unit, CHU Sainte-Justine, Université de Montréal, 3175 Chemin de la Côte Sainte-Catherine, Montreal, QC, Canada H3T 1C5

## Abstract

The monitoring of electrical activity of the diaphragm (EAdi) is a new minimally invasive bedside technology that was developed for the neurally adjusted ventilatory assist (NAVA) mode of ventilation. In addition to its role in NAVA ventilation, this technology provides the clinician with previously unavailable and essential information on diaphragm activity. In this paper, we review the clinical interests of EAdi in the pediatric intensive care setting. Firstly, the monitoring of EAdi allows the clinician to tailor the ventilatory settings on an individual basis, avoiding frequent overassistance leading potentially to diaphragmatic atrophy. Increased inspiratory EAdi levels can also suggest insufficient support, while a strong tonic activity may reflect the patient efforts to increase its lung volume. EAdi monitoring also allows detection of patient-ventilator asynchrony. It can play a role in evaluation of extubation readiness. Finally, EAdi monitoring provides the clinician with better understanding of the ventilatory capacity of patients with acute neuromuscular disease. Further studies are warranted to evaluate the clinical impact of these potential benefits.

## 1. Introduction

In the pediatric intensive care unit (PICU), up to half of patients require mechanical ventilation (MV) [1]. The objectives of this therapy are to support the failing respiratory muscles and allow better gas exchange while awaiting recovery. As simple a concept as MV may seem, it can be also detrimental and delay healing if not judiciously adjusted to the individual patient. Ventilation not synchronized with patients' efforts, as well as both insufficient or excessive support, can delay recovery, prolong the MV duration, and contribute to muscle wasting [2, 3]. It has long been considered crucial to monitor the respiratory activity of critically ill patients in order to limit these complications, but available monitoring methods were complex and rarely used in clinical practice [4, 5]. 

Over the last decade, a new minimally invasive technology has been developed to continuously record the electrical activity of diaphragm (EAdi) at bedside. The main purpose of this technology is to synchronize and adapt the ventilatory support following the EAdi signal during the neurally adjusted ventilatory assist (NAVA) mode [6]. In addition to NAVA ventilation, EAdi monitoring also provides the clinician with a comprehensible continuous evaluation of the diaphragm's function. The aim of this paper is to emphasize the importance of this new information in clinical practice.

## 2. Materials and Methods

### 2.1. Data Collection

This paper is based on both data from the medical literature and the authors' experience in the field. The authors are pediatric intensive care specialists with extensive clinical and research experience in pediatric mechanical ventilation and particularly in EAdi monitoring. In the last two years, we conducted more than 150 EAdi recordings in our PICU for clinical or research purposes. The extensive review of the medical literature on monitoring of the diaphragm and other respiratory muscles function in critically ill children was conducted using the PubMed research tool. The research strategy is described in Table 1.

### 2.2. Electrical Activity of Diaphragm Monitoring

**Figure 1:**
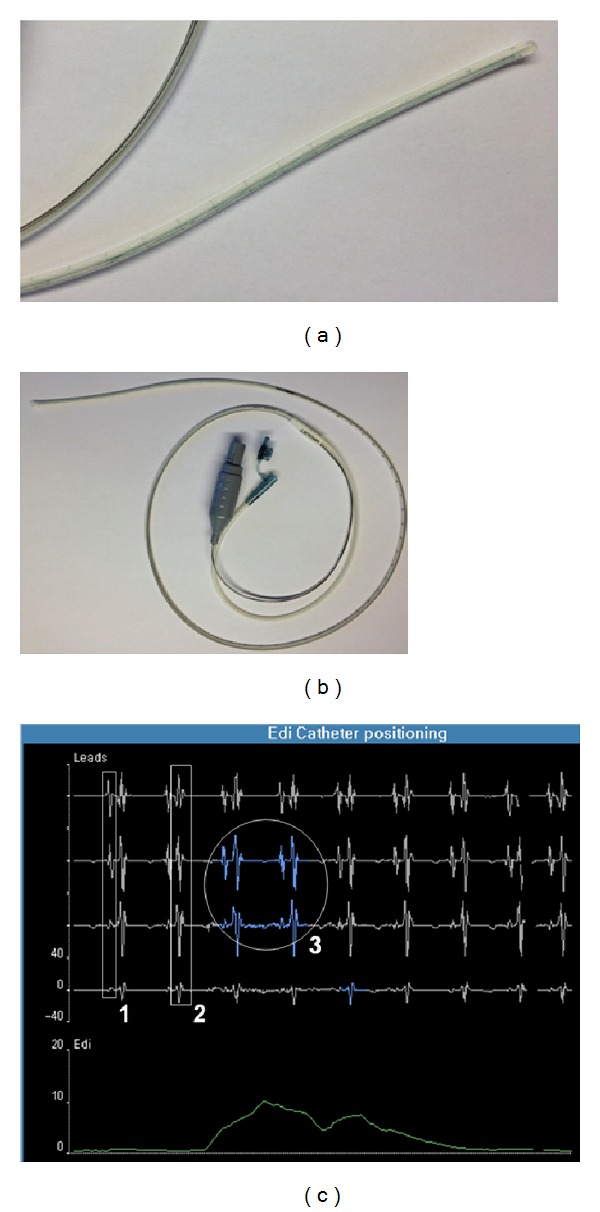
Specific nasogastric 8F catheter for EAdi monitoring (b), with an enlargement of the distal tip of the catheter equipped with microelectrodes (a). Screenshot of the specific interface for catheter positioning (c) with the three key components of the optimal position: (1) presence of P waves in the proximal lead with disappearance in distal lead; (2) decrease in the QRS amplitude from the upper to the lower leads; and (3) diaphragm electrical activity highlighted mostly in the central leads (in blue).

EAdi monitoring in our PICU is conducted using the technology provided by the servo I ventilator (MAQUET Critical Care, Solna, Sweden). The signal is obtained using specific nasogastric feeding tube (Figure 1) equipped with miniaturized electrodes placed at its distal end (NAVA catheter, MAQUET, Solna, Sweden). Crural EAdi signals are continuously processed with algorithms originally described by Sinderby et al. [7–10] to address the problems associated with the muscle-to-electrode distance and the influence of cardiac activity and esophageal peristalsis on the signal strength. The processed signal can be simply characterized by its amplitude on inspiration (peak inspiratory EAdi) and expiration (end-expiratory EAdi). The EAdi is controlled by the brainstem respiratory center and therefore reflects the respiratory drive [11]. 

In our PICU, as recommended by the manufacturer, the EAdi catheter is initially positioned using the “NAVA catheter positioning” screen on the servo I, as illustrated in Figure 1. This window screen displays four leads not filtered for ECG activity and the EAdi curve. Assessing the position of the microelectrodes relative to the heart allows for optimal positioning of the catheter, when the P waves and QRS complexes are large in the upper leads and smaller in the lower leads. The diaphragm activity highlighted in blue during inspiratory effort should appear mostly in the two middle leads. Respiratory therapists regularly check the correct position the same way. Nurses also regularly verify the position of the tube with its length marks. We consider this technology to be as minimally invasive as the nasogastric tube is standard of care in critically ill children with respiratory failure. 

## 3. Results and Discussion

### 3.1. Adjustment of Ventilatory Support with EAdi Monitoring

In the past decade, there has been growing concerns regarding the impact of prolonged intubation on diaphragmatic function. As little as 12 hours of full mechanical support can suffice to induce diaphragmatic atrophy [12]. Jaber et al. [13] observed similar findings on postmortem diaphragm biopsies of brain-dead organ donors, with a decrease in low-twitch and fast-twitch muscle fibers of 57% and 53%, respectively compared to a control group. This atrophy was associated with a proportional loss in diaphragm contractility [13]. In the pediatric population, data are more limited, but similar atrophy has been reported [14]. Oxidative stress induced by muscle inactivity seems to contribute to muscle atrophy [15]. Oxidative stress to actin and myosin triggers degradation of the myofibrillar lattice through the proteolysis pathway [16]. In addition, unloading the diaphragm reduces protein synthesis by 30% within the first 6 hours of ventilation and persistently so for the following 12 hours [17]. Diaphragm atrophy becomes clinically very relevant once the patient is ready to be weaned off the ventilator since respiratory muscles strength is key to a successful extubation [18]. These findings suggest that the maintenance of a diaphragm activity by avoiding full support ventilation may limit atrophy and lead to an earlier extubation [19, 20]. Even assisted mode can induce muscle atrophy if the level of support is excessive [21]. Determining the optimal support is therefore a clinical challenge. EAdi becomes an essential clinical device since it allows the physician to monitor the diaphragmatic efforts and adjust appropriately the ventilator settings protecting against potential atrophy.

**Figure 2:**
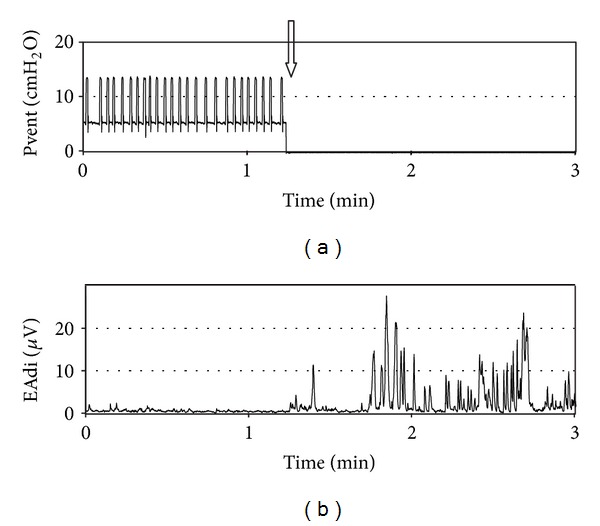
Evolution of ventilatory pressure (a) and electrical activity of the diaphragm (b) surrounding extubation (arrow) in a 2-year-old girl with meningitis. Note the very low diaphragm activity prior to extubation on low level of assisted ventilation (pressure support of 7 cm H_2_O).

EAdi variation in response to modification of respiratory assistance in conventional mechanical ventilation as well as in NAVA mode has been described by Colombo et al. [22]. Increasing ventilatory support decreases or abolishes neural respiratory drive, which may suggest overassistance, while decreasing ventilatory support increases EAdi, suggesting underassistance. In our experience, we were surprised to frequently observe abolished ventilatory drive in intubated children. Around 25–30% of patients with conventional ventilation have no measurable inspiratory EAdi. The majority of these observations were made in patients in whom ventilatory drive suppression had not been desired by the treating physician. Suppressed ventilatory drive can even be observed in patients with low levels of support, particularly in patients with normal lungs. This concept is well illustrated in Figure 2, which shows the almost absent ventilatory drive of a 2-year-old girl intubated for meningitis and ventilated with pressure support of 7 cm H_2_O. It contrasts well with the clear phasic EAdi after extubation. Periods of absent ventilatory drive have also been reported in neonates and children [23]. When clinically indicated, a decrease of the ventilatory support or a switch to NAVA usually leads to the reappearance of diaphragm activity. 

**Figure 3:**
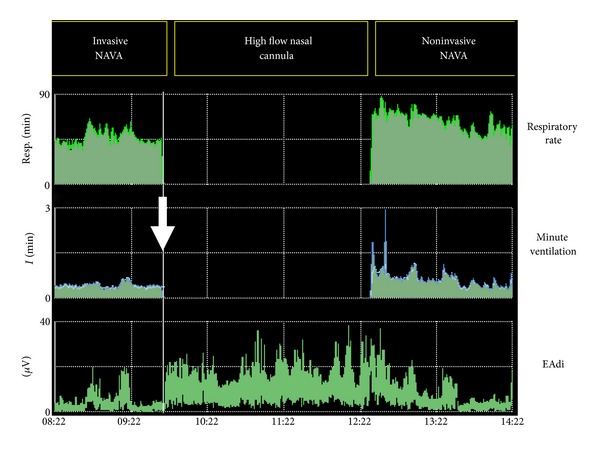
Evolution of respiratory rate, minute ventilation, and EAdi in a 15-day-old girl in the postoperative period of aortic valvotomy. After extubation (arrow), the infant was immediately supported with high flow nasal cannula. Progressive respiratory failure led to the introduction of noninvasive ventilation with NAVA 3 hours after extubation. An increase in EAdi was evident shortly after extubation, prior to the onset of clinical respiratory distress. The improvement of the respiratory failure with noninvasive ventilation was rapidly followed by a decrease in EAdi, toward preextubation levels.

On the other hand, very high inspiratory EAdi can be observed in patients with severe respiratory conditions (severe bronchiolitis, severe bronchopulmonary dysplasia, extubation failure, acute respiratory distress syndrome, and so on). Adjusting the support (e.g., increase of the support level, or introduction of noninvasive ventilation in case of post-extubation distress) usually succeeds in normalizing levels of EAdi.  Figure 3 describes the evolution of EAdi following extubation in a 15-day-old girl in the postoperative period of aortic valvotomy. The infant was initially supported with high flow nasal cannula, but progressive respiratory distress led to the introduction of noninvasive ventilation with NAVA 3 hours after extubation. Interestingly, the EAdi monitoring showed an increase in EAdi shortly after extubation, prior to the onset of clinical respiratory distress. The improvement of the respiratory failure with noninvasive ventilation was paralleled by the decrease in EAdi, toward preextubation levels. 

Noteworthy, certain patients (typically infants with severe bronchopulmonary dysplasia) continue to maintain elevated drive despite MV adjustment, demonstrating the severity of their lung disease. 

This shows how the level of ventilatory support can interfere with the child's respiratory drive. It is a challenge for physicians to detect both excessive or insufficient support based solely on clinical examination. This often leads to inappropriate assistance and increased risk of diaphragmatic atrophy. EAdi provides unique information to the clinician and allows better tailoring of the ventilatory support. 

### 3.2. Tonic Activity of the Diaphragm

**Figure 4:**
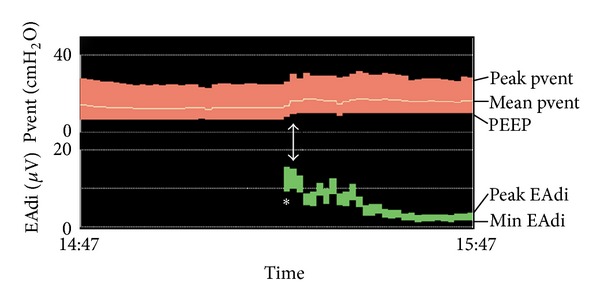
Example of very high level of tonic EAdi (*) observed in a 2-year-old boy with acute respiratory distress and hypoxemia. Tonic EAdi rapidly decreased following the increase in PEEP from 7 to 10 cm H_2_O (arrow) (see text for details).

Tonic EAdi is the diaphragm activity that persists until the end of expiration above baseline. Tonic EAdi is usually absent in normal adult or children older than one year [24]. In normal infants, the end-expiratory lung volume (EELV) is actively maintained above the relaxation volume to prevent derecruitment of the alveoli. Different mechanisms involved in this process include rapid respiratory rate with short expiration, braking of expiratory flow with larynx constriction, and persistence of diaphragm activity into expiration [25]. In intubated children, the endotracheal tube precludes the laryngeal braking, reinforcing the importance of tonic EAdi. Animal models have shown that conditions of abdominal distension, lung deflation, pulmonary edema, and acute lung injury can increase the tonic activity of the diaphragm [26–29]. In intubated infants, the suppression of PEEP also induces an increase in tonic EAdi [25]. Elevated tonic EAdi may increase the risk of respiratory fatigue by increasing diaphragm metabolism in situations of possible inappropriate perfusion [25, 30, 31]. Increased tonic EAdi may reflect patient efforts to increase his EELV. Adjusting positive end-expiratory pressure (PEEP) should be considered.  Figure 4 illustrates the case of a patient with severe acute respiratory distress syndrome secondary to a pneumonia in whom the use of the NAVA catheter allowed the physician to notice an unusual elevated tonic EAdi. The PEEP was consequently increased from 7 to 10 cm H_2_O, and this adjustment was followed by a rapid decrease in tonic EAdi and improved arterial saturation. This improvement in this case might also have been the result of other manoeuvers such as secretion suctioning and opening of atelectatic areas. However, the importance of normalizing EAdi cannot be undermined. 

### 3.3. Extubation Readiness Evaluation

Adequate diaphragmatic function is paramount to ventilator weaning [32]. Using EAdi monitoring to help predict extubation success has therefore been considered. The concept of neuroventilatory efficiency (NVE) represents the ratio between the tidal volume and the EAdi and therefore the ability of the diaphragm to generate a tidal volume normalized to the neural drive. In the only pediatric study done on extubation readiness, Wolf et al. [33] observed that the ability to generate a higher diaphragmatic activity for the same tidal volume in pressure support ventilation was a predictor of successful extubation. This may appear somehow counterintuitive, as the ratio of tidal volume to EAdi should reflect the performance of the neuromechanical coupling. However, it is important to mention that the ratio was measured during ventilatory support, the latter varying among patients. As previously mentioned, ventilatory support can influence children's ventilatory drive in very different ways accordingly to the disease. It would therefore be important to measure this ratio in absence of any ventilatory support, in order to precisely evaluate the intrinsic ability of the diaphragm rather than that of the ventilator. In a recent study by Liu et al. [34] on extubation readiness in critically ill adult patients, a higher NVE value during a spontaneous breathing test was observed in successfully extubated patients. On the contrary, patients in the extubation failure group had an EAdi increase twice that observed in the successfully extubated group. Furthermore, Wolf et al. also demonstrated a direct correlation between EAdi and negative inspiratory airway opening pressure [33]. The latter is a valuable measure of neuromuscular drive and extubation readiness [35, 36]. While EAdi seems to be a promising clinical tool to predict extubation readiness, further pediatric studies are warranted to identify the clinical predictive variables and to analyze their prognostic performance. 

### 3.4. Followup of Diaphragm Function in at-Risk Children

**Figure 5:**
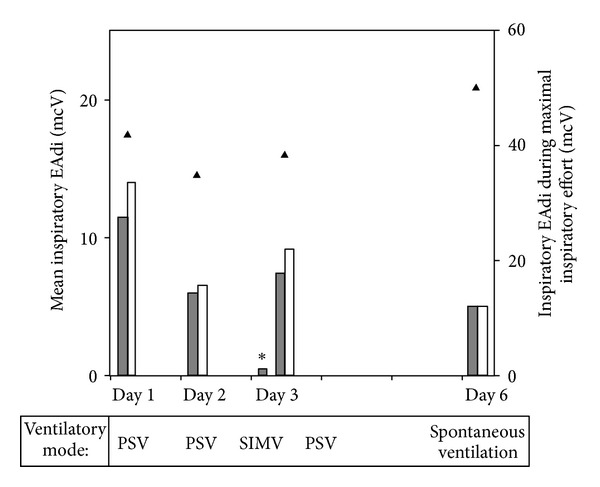
Daily evaluation of ventilatory drive in a 5-year-old patient with cervical myelitis. Each day, mean inspiratory EAdi was recorded during 5 minutes with the ventilation prescribed by the attending physician (grey bars) and 5 minutes under continuous positive airway pressure (CPAP, white bars). Inspiratory EAdi was also measured during 2 voluntary maximal inspiratory efforts (triangle). A period of overassistance with absent ventilatory drive was detected at day 3 (*). The recovery of the ventilatory drive was observed rapidly after adjustment of the support. (PSV: pressure-support ventilation; SIMV: synchronized intermittent mechanical ventilation).

In patients with acute neuromuscular disease, it is essential to be able to rapidly detect the worsening of the disease and its impact on ventilatory function. Clinical observation alone might be insufficient. A recent case report illustrates the clinical utility of EAdi monitoring in the particular setting of two infants with botulism [37]. In our PICU, we routinely monitor the evolution of EAdi in at-risk population such as patients with Guillain-Barré syndrome, infant botulism, transverse myelitis, or cervical spinal cord injury. In order to compare EAdi values and detect a true trend, it is important to measure EAdi under the same conditions. In extubated patients, EAdi is recorded at rest and during respiratory efforts such as coughing or deep inspiration. In intubated patients, we measure EAdi in predetermined periods of CPAP or low pressure support mode on repeated occasions. Moreover, with the continuous monitoring, the physician is able to recognize whether his patient is either over- or underventilated, which may be particularly detrimental in such conditions.  Figure 5 illustrates the daily evolution of EAdi parameters during the PICU stay of a 5-year-old patient with cervical myelitis. No clear deterioration of ventilatory drive was observed in this patient, but daily monitoring allowed the detection of a period of overassistance with absent ventilatory drive at day 3. Ventilatory drive recovered rapidly after adjustment of the support.

### 3.5. Detection of Patient-Ventilator Asynchrony

To maintain an appropriate respiratory drive, synchronization is key. Patient-ventilator asynchrony is defined as a mismatch between the inspiratory and expiratory phases of the patient and the ventilator. This includes the inspiratory and expiratory delays (time between patient demand and ventilator response), wasted efforts (efforts undetected by the ventilator), autotriggering (ventilator assist delivered in absence of patient demand), and double-triggering (two rapidly successive ventilator assists following a single effort). In adults, asynchrony is associated with prolonged ventilator support, longer stay in the intensive care unit, and total hospital length of stay [3, 38, 39]. Asynchrony is very difficult to appreciate by visual examination of the patient and evaluation of the ventilator waveforms [40]. In contrast, EAdi monitoring permits an easy detection of asynchrony. A few years ago a study from our center demonstrated poor synchronization between infants ventilated in the synchronized intermittent mandatory ventilation mode and their ventilator with conflict occurring 53 ± 26% of the time [41]. Similar results were shown in a more recent study, with asynchrony occurring 25 ± 10% during conventional ventilation [42]. NAVA mode which adapts support according to EAdi should be considered in patients with marked asynchrony if adjustment of conventional ventilator settings has failed. This strategy has been proven effective in adults [22, 43] and children [23, 42, 44–46].

### 3.6. Limitations of EAdi Monitoring

The EAdi technology is relatively new. Many of the concepts mentioned in this paper are theoretical and have not been confirmed by large studies. In particular, the impact of this monitoring on clinical outcomes (MV duration, extubation failure, and so on) remains to be validated. Like any new technology, it is important to balance its risks, costs, and benefits. So far, no complications have been detected. 

Another limitation to EAdi monitoring is the lack of reference values for both healthy and critically ill children. With experience, it becomes rather easy to interpret clearly abnormal values. More studies are needed to determine the optimal individual EAdi target. 

It is important to emphasize that EAdi represents respiratory drive and not diaphragmatic contractility. Diaphragmatic strength can be estimated by the volume or pressure generated by a given EAdi value (neuromechanical efficiency). This has not been studied in children. Contractile performance can also be tested using other more complex methods, for example, airway occlusion pressure generated by the diaphragm during phrenic nerve stimulation [47] and transdiaphragmatic pressure measurement during a standardized task.

Finally, this technology is simple to use but can also be misinterpreted. In particular, the appropriate position of the catheter should always be confirmed before interpretation of EAdi values.

## 4. Conclusions

In conclusion, EAdi monitoring is a very accessible bedside technology that provides relevant information regarding the patient's respiratory drive during critical illness. It allows the clinician to better adjust ventilatory settings to each patient's particular condition and protect against asynchrony and diaphragmatic atrophy. Further studies are needed to evaluate the impact of this new technology on clinical outcome.

## Figures and Tables

**Table 1:** Search strategy for identifying related articles in Medline.

No. 1	Pediatric OR paediatric OR infant* OR child OR children [All]
No. 2	Mechanical ventilation [All]
No. 3	Assisted ventilation [All]
No. 4	No. 2 OR No. 3
No. 5	Diaphragm electromyography [All]
No. 6	Diaphragmatic electromyography [All]
No. 7	Electrical activity diaphragm [All]
No. 8	Diaphragm* function [All]
No. 9	Respiratory muscle monitoring [All]
No. 10	No. 5 OR No. 6 OR No. 7 OR No. 8 OR No. 9
No. 11	No. 1 AND No. 4 AND No. 10

No limits were set for the time period or the language of publication.

## References

[1] Santschi M, Jouvet P, Leclerc F (2010). Acute lung injury in children: therapeutic practice and feasibility of international clinical trials. *Pediatric Critical Care Medicine*.

[2] Lemaire F (1993). Difficult weaning. *Intensive Care Medicine*.

[3] Thille AW, Rodriguez P, Cabello B, Lellouche F, Brochard L (2006). Patient-ventilator asynchrony during assisted mechanical ventilation. *Intensive Care Medicine*.

[4] Tobin MJ (1990). Respiratory monitoring during mechanical ventilation. *Critical Care Clinics*.

[5] Fitting JW (1989). Respiratory muscle function in the critically ill patient. *Acute Care*.

[6] Sinderby C, Navalesi P, Beck J (1999). Neural control of mechanical ventilation in respiratory failure. *Nature Medicine*.

[7] Beck J, Sinderby C, Lindström L, Grassino A (1996). Influence of bipolar esophageal electrode positioning on measurements of human crural diaphragm electromyogram. *Journal of Applied Physiology*.

[8] Beck J, Sinderby C, Weinberg J, Grassino A (1995). Effects of muscle-to-electrode distance on the human diaphragm electromyogram. *Journal of Applied Physiology*.

[9] Sinderby CA, Beck JC, Lindström LH, Grassino AE (1997). Enhancement of signal quality in esophageal recordings of diaphragm EMG. *Journal of Applied Physiology*.

[10] Sinderby C, Beck J, Spahija J, Weinberg J, Grassino A (1998). Voluntary activation of the human diaphragm in health and disease. *Journal of Applied Physiology*.

[11] Lourenço RV, Cherniack NS, Malm JR, Fishman AP (1966). Nervous output from the respiratory center during obstructed breathing. *Journal of Applied Physiology*.

[12] Levine S, Nguyen T, Taylor N (2008). Rapid disuse atrophy of diaphragm fibers in mechanically ventilated humans. *The New England Journal of Medicine*.

[13] Jaber S, Petrof BJ, Jung B (2011). Rapidly progressive diaphragmatic weakness and injury during mechanical ventilation in humans. *American Journal of Respiratory and Critical Care Medicine*.

[14] Knisely AS, Leal SM, Singer DB (1988). Abnormalities of diaphragmatic muscle in neonates with ventilated lungs. *Journal of Pediatrics*.

[15] Powers SK, Kavazis AN, DeRuisseau KC (2005). Mechanisms of disuse muscle atrophy: role of oxidative stress. *American Journal of Physiology*.

[16] Shanely RA, Zergeroglu MA, Lennon SL (2002). Mechanical ventilation-induced diaphragmatic atrophy is associated with oxidative injury and increased proteolytic activity. *American Journal of Respiratory and Critical Care Medicine*.

[17] Shanely RA, Van Gammeren D, DeRuisseau KC (2004). Mechanical ventilation depresses protein synthesis in the rat diaphragm. *American Journal of Respiratory and Critical Care Medicine*.

[18] Vassilakopoulos T, Petrof BJ (2004). Ventilator-induced diaphragmatic dysfunction. *American Journal of Respiratory and Critical Care Medicine*.

[19] Brochard L, Harf A, Lorino H, Lemaire F (1989). Inspiratory pressure support prevents diaphragmatic fatigue during weaning from mechanical ventilation. *American Review of Respiratory Disease*.

[20] Futier E, Constantin JM, Combaret L (2008). Pressure support ventilation attenuates ventilator-induced protein modifications in the diaphragm. *Critical Care*.

[21] Hudson MB, Smuder AJ, Nelson WB, Bruells CS, Levine S, Powers SK (2012). Both high level pressure support ventilation and controlled mechanical ventilation induce diaphragm dysfunction and atrophy. *Critical Care Medicine*.

[22] Colombo D, Cammarota G, Bergamaschi V, De Lucia M, Corte FD, Navalesi P (2008). Physiologic response to varying levels of pressure support and neurally adjusted ventilatory assist in patients with acute respiratory failure. *Intensive Care Medicine*.

[23] Alander M, Peltoniemi O, Pokka T, Kontiokari T (2012). Comparison of pressure-, flow-, and NAVA-triggering in pediatric and neonatal ventilatory care. *Pediatric Pulmonology*.

[24] Colin AA, Wohl MEB, Mead J, Ratjen FA, Glass G, Stark AR (1989). Transition from dynamically maintained to relaxed end-expiratory volume in human infants. *Journal of Applied Physiology*.

[25] Emeriaud G, Beck J, Tucci M, Lacroix J, Sinderby C (2006). Diaphragm electrical activity during expiration in mechanically ventilated infants. *Pediatric Research*.

[26] Meessen NEL, Van Der Grinten CPM, Folgering HTM, Luijendijk SCM (1993). Tonic activity in inspiratory muscles during continuous negative airway pressure. *Respiration Physiology*.

[27] D’Angelo E, Pecchiari M, Acocella F, Monaco A, Bellemare F (2002). Effects of abdominal distension on breathing pattern and respiratory mechanics in rabbits. *Respiratory Physiology and Neurobiology*.

[28] Ma A, Bravo M, Kappagoda CT (2003). Responses of bronchial C-fiber afferents of the rabbit to changes in lung compliance. *Respiratory Physiology and Neurobiology*.

[29] Gunawardena S, Ravi K, Longhurst JC (2002). Responses of C fiber afferents of the rabbit airways and lungs to changes in extra-vascular fluid volume. *Respiratory Physiology and Neurobiology*.

[30] Hussain SN (1996). Regulation of ventilatory muscle blood flow. *Journal of Applied Physiology*.

[31] Bellemare F, Grassino A (1982). Effect of pressure and timing of contraction on human diaphragm fatigue. *Journal of Applied Physiology*.

[32] Laghi F, Cattapan SE, Jubran A (2003). Is weaning failure caused by low-frequency fatigue of the diaphragm?. *American Journal of Respiratory and Critical Care Medicine*.

[33] Wolf GK, Walsh BK, Green ML, Arnold JH (2011). Electrical activity of the diaphragm during extubation readiness testing in critically ill children. *Pediatric Critical Care Medicine*.

[34] Liu L, Liu H, Yang Y (2012). Neuroventilatory efficiency and extubation readiness in critically ill patients. *Critical Care*.

[35] Kuhlen R, Hausmann S, Pappert D, Slama K, Rossaint R, Falke K (1995). A new method for P0.1 measurement using standard respiratory equipment. *Intensive Care Medicine*.

[36] Nemer SN, Barbas CSV, Caldeira JB (2009). Evaluation of maximal inspiratory pressure, tracheal airway occlusion pressure, and its ratio in the weaning outcome. *Journal of Critical Care*.

[37] Bordessoule A, Emeriaud G, Delnard N, Beck J, Jouvet P (2010). Recording diaphragm activity by an oesophageal probe: a new tool to evaluate the recovery of diaphragmatic paralysis. *Intensive Care Medicine*.

[38] Chao DC, Scheinhorn DJ, Stearn-Hassenpflug M (1997). Patient-ventilator trigger asynchrony in prolonged mechanical ventilation. *Chest*.

[39] de Wit M, Pedram S, Best AM, Epstein SK (2009). Observational study of patient-ventilator asynchrony and relationship to sedation level. *Journal of Critical Care*.

[40] Colombo D, Cammarota G, Alemani M (2011). Efficacy of ventilator waveforms observation in detecting patient-ventilator asynchrony. *Critical Care Medicine*.

[41] Beck J, Tucci M, Emeriaud G, Lacroix J, Sinderby C (2004). Prolonged neural expiratory time induced by mechanical ventilation in infants. *Pediatric Research*.

[42] Bordessoule A, Emeriaud G, Morneau S, Jouvet P, Beck J (2012). Neurally Adjusted Ventilatory Assist (NAVA) improves patient-ventilator interaction in infants compared to conventional ventilation. *Pediatric Research*.

[43] Spahija J, De Marchie M, Albert M (2010). Patient-ventilator interaction during pressure support ventilation and neurally adjusted ventilatory assist. *Critical Care Medicine*.

[44] Beck J, Reilly M, Grasselli G (2009). Patient-ventilator interaction during neurally adjusted ventilatory assist in low birth weight infants. *Pediatric Research*.

[45] Breatnach C, Conlon NP, Stack M, Healy M, O’Hare BP (2010). A prospective crossover comparison of neurally adjusted ventilatory assist and pressure-support ventilation in a pediatric and neonatal intensive care unit population∗. *Pediatric Critical Care Medicine*.

[46] Clement KC, Thurman TL, Holt SJ, Heulitt MJ (2011). Neurally triggered breaths reduce trigger delay and improve ventilator response times in ventilated infants with bronchiolitis. *Intensive Care Medicine*.

[47] Rafferty GF, Mustfa N, Man WD (2005). Twitch airway pressure elicited by magnetic phrenic nerve stimulation in anesthetized healthy children. *Pediatric Pulmonology*.

